# Medical students’ motivations for participating in an elective focused on social inequalities and health disparities

**DOI:** 10.1007/s10459-024-10313-7

**Published:** 2024-02-05

**Authors:** Shahna Arps, Krista McCarthy Noviski, Lauren Tucker, Ameisha Tutwiler

**Affiliations:** 1 Department of Sociology and Anthropology, University of Toledo, Toledo, OH USA; 2 Judith Herb College of Education, University of Toledo, Toledo, OH USA; 3 College of Medicine and Life Sciences, University of Toledo, Toledo, OH USA

**Keywords:** Medical education, Student motivations, Adult learning theory, Social inequalities, Health disparities

## Abstract

In this study, we examine students’ reasons for pursuing elective training focused on medical racism and systemic health inequities at a midwestern medical school. Data collection included semi-structured interviews with students who participated in an optional course focused on these topics. We analyzed their motivations, goals, and interests using reflexive thematic analysis and created three themes based on students’ responses. Theme (1) “pre-existing conditions” focuses on students’ knowledge, beliefs, worldviews and experience prior to the class. Theme (2) “enacting change” examines their desires to become effective physicians and improve medicine overall. Theme (3) “creating community” considers their preferences for a supportive and connected learning and social environment. We discuss the findings within the context of adult learning theory and Self-Determination Theory. The research provides insight about the overt and underlying factors that drive medical students’ participation in training focused on social inequality. We also share recommendations for curriculum development and future research based on the patterns we found in students’ discussions of their needs and expectations.

## Introduction

In recent years, there have been growing efforts to reshape medical education in ways that emphasize the importance of social determinants of health, systemic health inequities, identifying and addressing bias, and anti-racism in curricula (Acosta & Ackerman-Barger, 2017; Gonzalez et al., 2020; Ufomata et al., 2021). In a large part, medical students are driving curricular changes through their interests in these topics and activism (Forrest & Geraghty, 2022; Afolabi et al., 2021; Lemieux et al., 2021). However, even as more institutions adopt statements that recognize the need for anti-racism-related policies and curricula, educational standards and requirements vary substantially, and training is not mandatory in all programs (Fatahi et al., 2023).

In their review of current medical education curricula, Fatahi et al. (2023: S19) identified a number of barriers with regard to the implementation of compulsory anti-racism training including “institutional culture, resistance from some faculty and students, lack of buy-in from affiliated hospital systems, lower prioritization because of greater focus on preparing for board examinations, few faculty with deep knowledge and expertise, insufficient resources allocated, and perceptions of a ‘minority tax’” (see Rodríguez et al., 2015 for a discussion of the minority tax). Similarly, Warnock et al. (2023: 414–415) described how “administrative barriers,” including a lack of support from institutional leaders and inadequate “space” in their mandatory curriculum ultimately prevented their student-initiated anti-racism training from becoming a required component in their program, and instead, was offered on a voluntary basis. While elective training can help fill gaps in medical education, as they point out, approaches that make training optional rather than required fail to reach all students despite the widespread relevance of the content.

Furthermore, multiple barriers to medical student participation in elective programs focused on health disparities and bias have been identified in the literature, such as time constraints, feelings of shame or discomfort talking about difficult topics, the “hidden curriculum” (i.e., an institutional culture that tolerates bias), inadequate instruction by non-expert facilitators, and other structural issues (Gonzalez & Bussey-Jones, 2010; Gonzalez et al., 2019). Since electives are completed outside of the compulsory curriculum, these courses may bring students limited institutional recognition while also requiring them to focus on material they are less likely to encounter on board exams. Nevertheless, an expanding body of literature focused on course development and training initiatives suggests that students are, in fact, enrolling in and meeting the learning objectives of these programs (see Lord et al., 2023; Suresh et al., 2022; Gonzalez et al., 2020; Bright & Nokes, 2019; Kutscher et al., 2016).

Less well documented are the reasons why many medical students decide to participate in optional educational opportunities despite their demanding courseloads and other barriers. Since students choose to take these courses, they may be differently motivated than mandated learners. They likely participate out of interest and/or because they recognize the value and importance of the topic (referred to as autonomous motivation, see Deci & Ryan, 2000). In general, the literature suggests that voluntary learners have more autonomous motivation and demonstrate a higher motivation to transfer acquired knowledge following a given training (Curado, 2015; Gegenfurtner, 2016). Numerous studies have linked autonomous motivation to positive outcomes such as better academic performance and achievement, enhanced intellectual investment, peer engagement, and more reflection in learning (Kusurkar et al., 2013a; Kursurkar et al., 2013b; Azila-Gbettor, 2021; Sobral, 2004). As such, elective learning formats may have important benefits for classroom dynamics if the relationships between motivations and outcomes hold true for courses focused on social inequality. Without better understandings of learners’ motivations though, our ability to establish best practices for mandatory and elective training on these topics is limited.

Further studies can also advance our knowledge about how medical students view formal curricula, medicine, and medical systems. In the current literature, calls to action from medical student-authors for curriculum development focused on anti-racism and social determinants of health, as well as publications that describe and evaluate specific educational initiatives, provide some insight into student perspectives regarding the need for and importance of training. For example, as medical students, Nieblas-Bedolla et al. (2020) highlighted the problems associated with the often-imprecise use of race in medical school curricula (i.e., race as a “proxy for pathology”, 1802) as well as the overall lack of attention to how medicine has perpetuated racial inequities. They advocate for an interdisciplinary educational approach to draw on expertise in fields such as ethics, history, and sociology in order to train future physicians who can effectively provide care for patients from different racial and ethnic backgrounds. Afolabi et al. (2021) echoed these concerns and recommendations for addressing shortcomings in medical education. In this case, medical students at the University of California, San Francisco identified gaps and missed opportunities to discuss race in their curricula and engaged in collaborative efforts to raise awareness about and address medicine’s role in perpetuating racism. In efforts to improve the curriculum, medical students provided a course review, suggestions for educational content, and strategies for facilitating conversations about race. First on the list was a recommendation to include experts from other fields (such as sociology, critical race theory, and gender studies) and community members as teachers.

Studies that include analysis of qualitative data collected from students who completed educational opportunities focused on anti-racism, health disparities, and bias, are limited in number, but do provide some descriptions of learning goals and preferences from student participants’ point of view. For instance, with regard to health disparities education, Gonzalez and Bussey-Jones (2010) found that medical students valued nonphysician instructors (including classmates and community members) and a safe learning environment. Students also wanted learning to be focused on knowledge and skill development intended to mitigate health disparities, such as how to navigate the medical system, advocate on behalf of patients, communicate effectively, and identify the roles of bias among providers.

Gonzalez et al. (2020) reported that preclinical medical students similarly emphasized their desire for a safe learning space and respectful tone during discussions in evaluations of an elective course focused on implicit bias recognition and management. In addition, students stressed competence and skill development, which included familiarity with academic literature, research evidence, and appropriate vocabulary in order to help them educate others and improve their conversations about implicit bias. Students also appreciated course materials focused on popular culture, which they could relate to and engage with more actively.

The importance students place on acquiring applicable knowledge is also reflected in Leung et al.’s (2016) description of how medical students at the Warren Alpert Medical School of Brown University designed and carried out a preclinical elective focused on underserved patients’ health and health care challenges. Medical students developed the elective in response to their observed shortcomings with regard to caring for uninsured patients at a student-run clinic. Student evaluations of the course focused on the need for solutions to the problems that were discussed in class and ways to link class topics to clinical practice. Their qualitative feedback on the course also emphasized the benefits of engaging with peers and local experts to learn from different perspectives about topics that were relevant to the community.

Although these studies provide important information about student perspectives, to our knowledge, only one previously published study explicitly describes student motivations for participating in an anti-racism-related elective course based on qualitative data. Lynn et al. (2023) analyzed a student-initiated program at RCSI University of Medicine and Health Sciences in Ireland and discussed thematic data focused on students’ reasons for participation. The authors identified the three themes of “education”, advocacy”, and “seeking a safe space to discuss and learn” in students’ responses (Lynn et al., 2023: 5). Students sought further education for “professional development”, “general interest”, and “personal growth” reasons. With regard to advocacy, students noted their desires to improve and build on the current RCSI curriculum, as well as to address racial inequity in order to improve society and health care on a larger scale. Lynn et al. (2023) also discussed students’ perspectives on barriers to discussing race, of which, lack of confidence was most commonly reported. Many students described their lack of lived experiences as a primary reason for their lack of confidence.

In their brief description of online survey responses, Lynn et al. (2023:5–6) provide a starting point for building an understanding of students’ motivations in the literature. However, further research is needed to create a more detailed picture of the factors that compel students to pursue elective training. Examining their motivations and goals is key to understanding students’ perceptions of medical education, as well as for developing effective curricula that meet their needs and expectations. In addition, an analysis of students’ viewpoints can contribute to adult learning theory, especially with regard to defining how medical students’ experiences, worldviews, and goals influence their educational pursuits.

To build on previous work, this study analyzes qualitative interview data about medical students’ reasons for pursuing elective training focused on social inequalities and health disparities. In doing so, we aim to evaluate what medical students’ responses can tell us about their views of medicine and medical education and examine how our findings can inform larger discussions of best educational models and practices. In the following sections, we describe the methods of data collection and analysis, discuss major themes in students’ detailed responses, compare the results with other published studies, and explain how students’ perspectives can inform effective course development and future research.

## Methodology

### Theoretical approach

This study is guided by adult learning theory, which encompasses a wide range of perspectives and models (Merriam, 2017). Research focused on adult learners suggests that they differ from child learners with regard to their motivations, experiences, and preferences (Knowles, 1980, 1984). We expect that medical students display typical adult learner characteristics as defined by Malcolm Knowles (1980, 1984): their learning is more independent and self-directed, they possess growing life experiences to draw on during learning, their inclinations to learn are problem-centered and related to their social roles, internal motivators for learning are often more important than external factors, and the reasons for learning must be clear, with an emphasis on immediate application. Indeed, the studies discussed in the previous section (Gonzalez & Bussey-Jones, 2010; Gonzalez et al., 2019; Leung et al., 2016; Lynn et al., 2023) provide evidence for the importance of life experience, internal motivators, and application-orientation among medical students who participated in anti-racism and health equity-related training. Furthermore, the emphasis on independence and self-direction among adult learners in humanistic theories is particularly important to this research, given that medical students are making their own decisions about pursuing elective training, rather than simply participating in the required curriculum. According to Self-determination theory (SDT), a theory of human motivation, autonomy is one of three critical psychological needs that influences motivation (Deci & Ryan, 2000). When medical students believe a learning activity to be personally interesting and/or valuable and they have a sense of choice and agency with regard to learning, this need for autonomy is met. When autonomy is combined with the need for competence (a desire to gain more expertise and master a subject) and relatedness (feeling connected to others in goals and purpose), overall motivation is higher and students are most likely to benefit from the activity (Deci & Ryan, 2000).

By enrolling in supplemental training, medical students may also be seeking opportunities to transform their perspectives in order to bring about more critical, autonomous, and responsible thinking (i.e., transformative learning, see Mezirow, 1991, 1997). As Merriam (2017:25) explained, “Learning in adulthood is often more than just adding information. It is also making sense of our experience and can result in a change in a belief, attitude, or perspective.” Elective topics such as bias, racism, and health disparities align particularly well with Mezirow’s (1991) transformative learning model, which emphasizes critically reflecting on assumptions that limit learners’ views of the world, revising meaning structures, and then acting on the basis of the transformed perspective.

Individuals’ decisions to participate in courses focused on anti-racism and health equity that foster reflection and transformative learning are also occurring within larger social and political contexts that deserve attention. Adult learners’ motivations for participating in education relate to perceived congruency between the prospective student and other students, teachers, and the larger institutional environment (Boshier, 1973). Learning, after all, is often a social activity. Adult learners, in particular, value collaborative and communal learning as they participate in and are guided by “communities of practice” (Wenger, 1999). In other words, the anticipated benefits of participating in a learning activity may include not only gaining new skills, but also access to a knowledge community with similar interests and goals, that can fulfill a sense of relatedness. As such, students may foresee multiple positive outcomes of their involvement; and Tough (1979) suggests that adult learners are conscious and capable of explaining the perceived rewards that drive their own behavior. Moreover, participation in a learning activity may be best understood as a chain or flow of responses rather than a single event or decision (Cross, 2003). In Cross’ (2003) model, the multiple and interacting variables that ultimately determine participation include self-evaluation of knowledge and skills, attitudes toward education (based on past experiences and social group influences), importance of goals and expectation that participation will meet goals, life transitions, opportunities for and barriers to participation, and perceptions about accurate information.

These different and often complementary perspectives relate to our research question about why medical students participate in educational opportunities outside of their mandatory curriculum and inform our methods of data collection and analysis. Adult learning theories not only help us understand students’ perspectives in this case study, but applying them to professional health care education also has the potential to improve learning outcomes through the development of educational strategies and programs (for reviews of adult learning theories and health care education, see Taylor & Hamdy, 2013; Mukhalalati & Taylor, 2019).

### Sample

All (52) medical students who participated in an elective course focused on medical racism and systemic health inequities at a midwestern medical school were invited to take part in the research. The elective course served as a way to identify students who not only reported an interest in studying social inequalities, but demonstrated this interest by actually becoming involved as student learners in the optional course. At this particular medical school, students voluntarily choose whether to participate in preclinical electives; they are not required to complete any elective hours. The course from which study participants were recruited was student-initiated and focused on a variety of topics related to social inequality and medicine. In addition to taking on typical student-learner roles, some participants also served as class coordinators and contributed to syllabus development, designing learning goals and assessments, and organizing lecturers for class sessions.

This specific elective follows a transformative learning approach (Mezirow, 1991) by encouraging medical students to identify and confront internal biases which cause them to enact racism, ableism, sexism, homophobia and fatphobia and ultimately change their behaviors and approaches to become anti-racist physicians. The course also focuses on the intersectionality of these biases through understanding economic and social theories. The class is organized into four segments to evolve from a broader societal review to individual reflection of the factors involved in racism and bias (i.e., foundational course sequencing). The four segments include (1) the history of racism, (2) the history of racism in medicine, (3) “racism and me” (focused on examining individual psychology and behavior), and (4) anti-racism. Class meetings are structured around presentations from individuals with relevant expertise (e.g., in history, political science, law, sociology, genetics, medicine, service provision, policy, and lived experience) as well as student discussions (small and large group) designed to encourage non-confrontational and non-coercive dialogues amongst peers. The inclusion of social media (e.g., TV show excerpts) is also used to promote discussion and critical reflection. Scenario-based activities, in which students share how they would act in certain situations followed by course leaders and lecturers responding with their input for discussion of new behavioral options, occur in the latter sections of the course. Students are expected to complete pre-work (assigned readings, videos) and journal reflections, attend class, and actively participate in class discussions and case analyses. The course is about a six-month commitment, with two-hour meetings once a week.

The initial idea for studying participants’ perspectives on the course was developed by the medical students who proposed the class. They recognized the importance of students’ viewpoints for evaluating curriculum development, as well as the need for support from experienced social scientists to carryout such an endeavor. At that point, they reached out to Arps (an anthropologist) and McCarthy Noviski (a sociologist) to collaborate on designing and conducting a multi-year qualitative research project. The study described in this article is part of that larger project. As researchers who contributed to the work focused on learners’ motivations, we positioned ourselves differently - as insiders (Tucker and Tutwiler), who are medical students involved in anti-racist education, and as outsiders (Arps and McCarthy Noviski), who have neither attended nor worked as faculty at a medical school. However, as a group, we approached this research with shared assumptions that educational initiatives focused on anti-racism and health equity are critical for medical education.

### Data collection and analysis

The University of Toledo’s Institutional Review Board approved the protocol for this study. We (Arps & McCarthy Noviski) collected data during semi-structured interviews that were held in-person or virtually based on the participant’s preference. All participants provided informed consent prior to the interview, which generally lasted about one hour. The interviews included open-ended questions about students’ motivations for enrolling in the course, why they chose to study systemic health inequities, bias, and medical racism, their specific interests, goals, what they hoped to learn, and the importance of these topics to their education, future training, and career. No incentives for participation were given. We presumed that participants would feel relatively comfortable freely stating their opinions during interviews, since the interviewers were not directly involved in course management or connected to the medical school. However, the formality of interview (made evident by consent procedures, office meeting contexts) and the knowledge that their responses would be used for research purposes may have impacted how students interacted with us and chose to answer interview questions.

We used an inductive and iterative approach in the reflexive thematic analysis of data (Braun & Clarke, 2019). While our analysis was data-driven, adult learning theory did inform our expectations about medical students’ learning characteristics. Also, each member of our research team viewed the data collected through unique lenses developed from our personal, educational, and career experiences. Together, our varied perspectives/subjectivities (as teachers and students, as new researchers and more experienced researchers, as members of minoritized and privileged groups) helped us interpret the meanings in students’ interview responses, including what was said explicitly as well as key, underlying messages. At the same time, we critically reflected on our own assumptions and experiences throughout the research and strived to remain open to differing points of view.

We gained familiarization with the data during the transcription process (simultaneous listening, writing out, and reading interview content), as well as through reading and re-reading transcripts and taking and reviewing notes about the transcripts. The first author (Arps) coded interview data, gradually constructing codes, refining them progressively in a circular, rather than linear way (going back to individual cases and recoding as necessary, integrating and connecting different codes across the dataset). The second author (McCarthy Noviski) reviewed the transcribed and coded data to facilitate reflexivity and deepen interpretation of data. Although the first author (Arps) took the lead in data analysis, all of us discussed findings throughout the project, sharing key insights about overt and implicit meanings across students’ responses that we developed during data collection, transcription of interviews, and the coding process. The first author (Arps) used diagramming to interpret connections between codes and create overarching themes and labels for the themes. Themes were shared with the other authors for their feedback and revisions. Following the example of Campbell et al. (2021), we chose to construct themes at both the descriptive/semantic and interpretive/latent levels in order to tell a meaningful story about students’ motivations for participation based on the data.

## Results

Twenty-one students volunteered to participate in the research, from two cohorts of the class (40.4% of total students who participated in the class). Fourteen participants were interviewed virtually (66.7%) and seven, in person (33.3%). Most participants were first-year medical students (14, 66.7%), six were in their second year (28.6%), and one was a third-year student (4.8%). During the interviews, participants described their varied age, gender, racial/ethnic, and socioeconomic backgrounds. Some of the self-reported identities of students included Black, African American, Asian American, Latino, Indian, Jewish, White, immigrant, abled, disabled, cisgender, man, woman, straight, gay, and queer.

The participants discussed multiple goals and reasons for studying social inequalities and health disparities in the elective course. In their responses, they highlighted the importance of competence, applicability, and engagement as motivations for participating in the class. We constructed three major themes with regard to underlying patterns in the data that tell a deeper story about these semantic labels: (1) pre-existing conditions, (2) enacting change, and (3) creating community. In the following sections, we describe these themes and include examples of participants’ responses to illustrate what their multiple voices tell us explicitly and implicitly about students’ views, experiences, and goals.

### Theme 1: pre-existing conditions

Students chose to take the class in order to learn about how racism and other social inequalities apply to medicine, historically and presently. The first theme, “pre-existing conditions,” helps us explore why students perceived a need for competency in these areas. Specifically, their voluntary participation was driven by a recognition of certain “pre-existing conditions.” These pre-existing conditions included a wide range of contextual factors, including a biased and unequitable medical system, self-identified gaps in their own knowledge, and their assessments of the formal medical curriculum.

Prior to enrolling in the class, students already realized that they were training to be a part of a medical system characterized by inequality. The class offered a way to better understand that system and make sense of their observations. For example, one student explained:

*I’m in school to be a doctor. So, I want to actually actively focus on the system that I’m about to train to be a part of, what the background and what the history is, and what people in the community of people who are interested in seeking equity, what they talk about when they talk about medicine and medical racism… I just want to apply a knowledge of there are higher things at work here. There are systems that have contributed to what I’m seeing before me. (14)*

In this response, and the one below, we also see students’ perspective that the current medical system is a product of large-scale historical forces. Also, the biased medical system is embedded in, and reflects, an unequal society.

*[I] need to learn and want to learn about issues like systemic oppression, racism, inequality; and there is definitely a huge need in the medical field to learn about those topics because they come up in medicine. They’re a part of medical history. (2)*

Underlying their descriptions of these pre-existing conditions is the idea that unequal social arrangements are unjust and perpetuate harm. As such, students viewed acquiring a better understanding of how social and systemic factors influence health as a foundational and essential skill, not just for them personally, but all medical students. One student explained:

*In an ideal world, I think all medical students should have an understanding of the history of our profession…those kinds of things* [racism, social determinants of health] *that if they’re not addressed, they get to the point where we’re at today, where people of color die earlier than White people and have poorer health outcomes. (7)*

To students, learning about medicine required attention to the legacies of racism and inequality, which are built into the structure of the health care system. In this way, students viewed health disparities and other failures of medicine as pervasive, systemic problems, rather than the result of isolated instances of individual bias. The following excerpt illustrates this perspective.

*I grew up in an affluent suburb, so I was often very shielded away from these real inequities. But I was also an Asian person in a predominantly White neighborhood. And I was affected…by systemic stuff…I grew up disabled. I did not get a lot of accommodations. So, it was quite difficult. And I think I had to realize very quickly, that it wasn’t individual people being cruel that was preventing my progress in anything. It was because the system was not designed for people like me. And that’s how you have to view things. Or else you’re just going to hate everyone forever. So, in that way, I realized, we have to tackle the big picture. (8)*

The statement above also shows how the theme of pre-existing conditions can help us understand why students had come to “realize,” prior to the class, that health inequities are systemic. Students knew about social inequality and medical biases through their own personal, educational, and work experiences. That is, the students themselves had pre-existing conditions, influenced by the circumstances of their lives, which they brought with them to the class. In terms of their personal identities, students discussed how their worldviews were shaped by encounters related to their race/ethnicity, immigrant background, religion, sexuality, and disability. In the following three excerpts, the students linked their awareness of and motivations to study systemic health inequities to their own firsthand experiences of bias and racism.

*I think a lot of it* [interest in the topic] *comes from me being a Black woman and seeing how things operated, just seeing how the system operated. Where were the deficiencies in the system? What was going wrong? I went to private school with mostly White people, so I was always the one Black girl. And so, I had a lot of experience with the thought processes of White people to be able to say, OK, this is where they’re willing to go with things and this is the problem, just because of my exposure. (10)*

*Growing up Indian, for example, my entire worldview is totally different than somebody who grew up White or somebody who grew up Black. Although in medicine, there’s a lot of Indian representation, within America itself, we’re still going through, there’s still a lot of racism. There’s still a lot of different microaggressions and things like that…Growing up, I didn’t see other people that looked like me, I was darker-skinned…I was bullied because of the way I looked. And other people didn’t have to go through that. So, it definitely changes your view on racism in general. (16)*

*I’m queer. My partner is a person of color who hasn’t been to the doctor in ages. He’s also Puerto Rican…so it’s like a whole thing that has a history of racism, medical racism, medical experimentation, exploitation. So, it’s kind of personal…to my partner and our relationship. (7)*

Similarly, multiple students described firsthand experiences of receiving poor quality medical care or observing issues with family members’ treatment, which they felt were related to their identities. For example:

*I’ve always had an interest in more of the humanities perspective when it comes to health care…Just personally, I’ve had a lot of adverse experiences in health care. My family has had a lot of adverse experiences as minorities in America. (3)*

*As a Black individual going out into the medical world, me having a voice and speaking up for my patients is something that’s really important to me… I have had medical experiences that were not the greatest. And it just makes you question, is it because I’m a woman and they’re not believing my symptoms? Or is it because I’m Black and a woman and they’re not believing my symptoms? Or is it just because maybe they’re not a great physician or all of these different things? But that’s always the question, why is it that they’re not believing what I’m saying? (21)*

Underlying these two excerpts is the awareness that inequality has persisted over multiple generations of family members (again pointing to the importance of long-term, historical forces) and that different aspects of identity (race and gender) potentially intersect to bring about poor treatment in medical contexts. Students with these lived experiences described being able to learn about these topics in class as particularly meaningful given their personal and familial experiences.

In other cases, the important pre-existing condition that motivated participation was ignorance. These students discussed their lack of lived experience related to racism and social inequality as the reasons why they believed they needed to pursue more learning on these topics. In particular, students brought up their affluence (problems like poverty and lack of medical insurance were unfamiliar to them), growing up in conservative, non-diverse communities, as well as the White, cisgender, straight, and male privilege described in the example below.

*I, being a cisgender, straight, White male just want to sit back and hear from as many different perspectives as possible, just because I don’t have any experience on this. And I’ve just wanted to learn more about the real-life experiences of people with different disabilities and different races and just how they are approached by medical care. (13)*

Students in this group also recognized how different aspects of identity intersect, and in the excerpts below they draw attention to overlapping types of privilege (Whiteness and affluence).

*If I’m interested in health equity, public health initiatives, and racial health equity as a White person, how does that function if I’m a privileged doctor working in a neighborhood that doesn’t, you know, have economic advantage or primarily is not White? And so those are the kind of questions that I wanted to learn more about and learn more about the history. (7)*

*I come from a lot of privilege, from a pretty rich, predominantly White suburb… generally didn’t have a lot of exposure to social determinants of health, like in a lot of low-income communities and honestly, communities of color. (14)*

Despite their acknowledged privileges, these students were previously exposed to social inequality and health disparities in secondhand, but impactful ways during different learning experiences. Both privileged students and those from minoritized backgrounds emphasized how their worldviews developed concurrently with their education and career paths. In terms of paid and volunteer work, they described jobs in AmeriCorps, hospitals, community health clinics, national institutes, and other positions that included diversity, equity, and inclusion (DEI), implicit bias, and bystander intervention training. Students described both positive and negative experiences as being influential. Although the elective was designed as a preclinical class, as the examples below show, some of the students did have experience working in health-related jobs.

*I worked in health care* [before medical school]. *I think there were only, in the two or three years that I worked as a nurse aide, I think I could probably count on both hands how many people of other races besides White I actually took care of, which is very surprising. And being in those scenarios, kind of just seeing how those patients are talked about and how they’re addressed by physicians and by the nursing staff and everything…I just wanted to learn more about everything. (13)*

*I remember in the ER seeing a lot of African American females who were scared when I worked as a scribe. Who were scared, who had pain, and all those scenarios. I saw them in the ER and then…the doctor would be like, “oh, this patient has fibromyalgia,” which was a “term,” like when they kind of exaggerate the pain. And I didn’t understand. I knew something was wrong…and really the issue for me at that time was the lack of knowledge. (6)*

*I actually learned a lot on the job [at a national institute] that the sentiment that race is a real biological determinant was also baked into genetics… So, I was like, well, I’ve got to do something about this, because this is really bad. (8)*

These excerpts show how students became aware of pervasive “pre-existing conditions” in their workplaces and started to question biased patient care and assumptions about race. Their observations pushed them to learn more about systemic health inequalities.

Similarly, students described previous educational experiences that shaped their views about society and medicine. Some were inspired by what they learned in undergraduate and graduate courses (often mentioning the importance of classes in sociology, anthropology, history, psychology, and gender studies) which provided foundational knowledge. Others emphasized that their previous education had been inadequate. They also discussed informal educational opportunities (such as speaker series), strategies to self-educate, as well as their positions in student organizations and/or advocacy groups. The example below shows how one student developed system-level thinking prior to taking the elective.

[During undergraduate studies], *I wanted to take a sociology course…and that’s why I got into learning about systemic things. I think the course director specified how systemic racism was causing discrimination of jail populations and how that’s a whole issue. And then after doing that, I’m like, man, it’s a lot bigger. It’s not just racist people. There’s a racist system that’s perpetuating those things. (1)*

While being exposed to these issues before the class, students acknowledged that their knowledge was still limited in some way; and they aspired to better understand humanity beyond their own life experience. They viewed this as an unmet need in their learning of medicine prior to the elective. They chose to further their education in response to the perceived limitations of the formal medical curriculum in preparing them to practice in a biased medical system. As such, we interpret student assessment of the required medical curriculum (design, content, and instruction) as yet another important pre-existing condition that ultimately motivated their participation in the elective. As we see in the following example, taking advantage of the extra, optional learning opportunity was an important way to supplement the required plan of study.

*I felt like we didn’t really get a lot of diversity talk anywhere else in our curriculum. So, I figured if I could take it* [the elective course] *and try to learn as much as I could, it would be better moving forward. (21)*

Similarly, in the excerpt below, a student describes the prioritization of foundational science topics in comparison to the time allocated for integration of the humanities and social sciences into medical curricula.

*Issues like systemic oppression, racism, inequality…they’re not often taught. They’re not often incorporated into medical education at the foundational level. They kind of get overshadowed by learning the fundamental basic sciences and we don’t have a whole lot of time to learn about the humanities side of medicine, which is, I think, the larger part of medical training. (2)*

In addition, multiple students mentioned that although race is discussed as a risk factor for disease (they often learned to associate diseases with particular racial groups in their required courses as a strategy for taking board exams), they sought additional opportunities to examine race and racism in medicine more critically and comprehensively. In particular, they pointed out the need for discussion of race as a socially-constructed concept, rather than a genetically meaningful way to categorize and treat people. In the following example, a student shares their concerns about the potential outcomes of these learning strategies.

*There’s so much content, we are taught fast and easy ways to group things together and to think of certain patients…You’re being taught, “OK, if someone is White, here are the diseases they’re more likely to have. If someone is Black, here are the diseases they’re more likely to have,” you know? And you’re taught those things so that when you’re reading a patient vignette, the very first thing you notice is the race and then you’re already eliminating a bunch of different possibilities. I understand why they tried to put it that way just because there’s so much material that it’s impossible to make every little consideration. But then those things are super harmful when you apply them to real life. You start to miss a diagnosis because someone has a disease that you didn’t consider because they’re not White…Maybe it’s a small percentage for you, but like for that one patient, that’s their entire life, that’s their health that is ruined because someone wouldn’t consider this on their differential for the patient. (12)*

Others also discussed how research in the humanities and social sciences applies to their training in patient-centered health care and emphasized that instruction of these topics in the curriculum should come from experts in those fields, as well as from people with lived experience (with regard to encountering a biased medical system and/or service provision in minoritized communities). As one student commented:

*You can’t ask random PhDs and MDs to teach this, right? The issue is one of qualification… I have [an advanced degree]. I have a copy of Harrison’sInternal Medicine*. *It doesn’t mean that I’m qualified to teach pulmonology. You can’t assume that any person can teach this stuff. (9)*

Students did not want to see the additional teaching burdens fall only on minoritized faculty or students either (i.e., “the minority tax”); and they recognized the substantial labor that went into developing and running the elective course that is the focus of this paper. In the excerpt below, a student who stressed the importance of having access to the class expressed this view:

*It shouldn’t be incumbent on Black [students] to educate other students on what medical racism is, because it’s not their responsibility. (7)*

Students chose to participate in the elective because it offered the opportunity to learn from a number of different presenters who, because of their training and experience in a variety of fields, could help provide a more complete picture of course topics.

There is likely an additional, more latent reason for students’ emphasis on formal classroom training in these topics. Students “knew” that social and systemic factors influence health, experientially and intuitively, but they strived for a more academic-focused competency, too. This is likely linked to their perceptions about how to gain authority in a system that ostensibly values objectivity, facts, and scientific empiricism. One student explained:

*I wanted to know more about the specifics, because I need the specifics to be able to talk about it, to present myself as an informed source, a reliable source of information for other people. (3)*

In the student’s comment we see the perspective that preconditions for being recognized as an informed, reliable “expert” exist. To be taken seriously, that individual must be able to present “unbiased facts” to back up any potential critiques of the medical system. As an academic course, students expected that the elective would provide this type of training, even if it did not come with the same recognition as a required component of the curriculum.

### Theme 2: enacting change

Students’ descriptions of their goals and motivations for participation in the class emphasized action and positive change. They were not just interested in understanding the root causes of health disparities, they sought solutions to the problems– namely, how to provide effective patient-based care in a biased medical system embedded in an unequal society and how to transform that medical system and society to achieve health equity (i.e., pre-existing conditions discussed in the previous theme). As such, both advocacy and activism were goals motivating participation in the course.

In general, students’ reasons for taking the course aligned with their larger career goals and values. They were working to become doctors, not just to make a living but to make a difference in people’s lives and communities. For example:

*It’s not like I’m just going to get in here, do my work, make money, and retire. You want to leave it a better place than you found it. (19)*

*Because the end goal is to just improve the quality of lives of your patients, their community, and just people as a whole. (5)*

Students hoped that learning about racism and systemic health inequities, both historically and presently, would help them become empathetic and informed doctors. In particular, they emphasized the need to understand systemic barriers that patients from different backgrounds face in order to better serve them. In the following excerpt, a student demonstrates respect for the humanity of potential patients and recognition of the physician’s power.

*If I become a physician, I need to understand the area I’m working in, what population I’m going to be working with. I need to understand their needs. I need to understand their backgrounds. I’m not just a clinician when I go there. I need to understand them as humans…I realize how impactful being a physician is to another person’s life, and it’s a huge responsibility on our shoulders and I need to do a lot of learning before I get to the clinical world. (6)*

The example above, as well as the two below, are representative of a larger pattern across the dataset, where students (regardless of their personal backgrounds) focused on the likelihood of encountering substantial diversity in their future patients. In order to meet their needs effectively, they needed to learn more about different perspectives and health care experiences and critically analyze their own biases and assumptions in order to practice cultural humility.

*No matter where you are, you’re going to eventually come in contact with someone that’s not from your background, not from your race, and you still need to be able to provide care to them and know how to speak to them. (18)*

*Identification of personal implicit biases that you might have is particularly useful information, information you need to have if you’re going to conduct yourself in a situation where you’re going to be interacting with a large variety of people, a very diverse group of people. They’re entrusting their lives in you. So, you’ve really got to make sure that you’re giving them the full attention and care that they need. (15)*

In the following excerpt, the student acknowledges the challenges of understanding someone else’s situation, and advocates for letting the patient (rather than the physician) determine their own needs.

*Very rarely are your patients going to be like you… I think it’s really important to recognize that, and sort of be able to engage and challenge yourself with being uncomfortable because you don’t fully understand what somebody else’s situation is like. And I think it’s extremely important to be able to do that, to meet your patients at their level. And meet their needs in a way that suits them. (5)*

This rejection of a paternalistic approach to health care is just one way students expressed their resistance to a stratified medical system in which doctors, rather than patients, are viewed as having all of the relevant knowledge and experience about health. Students emphasized being committed to a collaborative, rather than coercive model of care.

Students also discussed how their decisions to participate in the course aligned with their aspirations to help create a better, more equitable society and medical system. Learning how medicine reflects society, particularly in terms of structural inequalities, would allow them to better participate in developing solutions to current problems (i.e., they valued problem-based learning). Beyond providing high quality care to individual patients, they sought strategies for bringing about large-scale change by influencing policy and engaging in other forms of advocacy. In fact, some students reported that they specifically plan to focus their career on health equity. For example:

*I think I got into health care mainly for health equity. Seeing people like my neighbors not being able to get health care, that’s super messed up*, [especially] *being a country that has the means to deliver health care to its population… I’m interested in fixing the problems of our time, which in my mind, this* [inequity] *is one of the top on the list, if not the top. So, I’m trying to figure it out. It’s hard as hell. (7)*

The excerpt above, as well as the one below, show how students are critical of the status quo, and reject the notion that they are powerless to change it in spite of the challenges involved in doing so.

*I think that racism in medicine, as in the rest of society, is a huge problem. And I don’t think big problems get solved by people just hoping that they do. And so, I thought that this* [course] *would be a way to contribute…to do something to make at least my part of the world a marginally better place.…And I also just do not accept that there’s nothing to be done. (9)*

During interviews, students conveyed excitement, passion, anger, and resolve that, along with their words, demonstrated their beliefs that large-scale change was necessary and possible. A key thread underlying this theme is that students viewed the course as empowering. For example:

*I’d rather keep pushing boundaries than just be sitting back and not helping improve medicine because, I mean, I’m going into this for a reason. I’m going into medicine because I love it and I want patients to get the best care. (16)*

Students also described their involvement with student organizations (for example, White Coats for Black Lives) and volunteer work serving marginalized communities that overlapped with the course focus on social inequalities and health disparities. In other words, the course was viewed as an integral part of their overall engagement with social justice issues. Students were also motivated by the activism they observed within recent social movements and often discussed protests against racism, particularly those that gained global attention in 2020, the Black Lives Matter Movement, and health disparities brought to light during the COVID pandemic. They described how social movements demonstrated the relevance of the topics to be discussed in the elective class and inspired them to learn more. Specifically, students connected what they were seeing on the news, social media, and in communities around the US to their education and goals to promote change. For example:

*I was in Seattle, Washington, so a very progressive city, a place where a lot of people are talking about these larger social issues and it was during the Black Lives Matter protests. So, being all wrapped up in the movements of the time really made me start to think about these things. (14)*

*Go back all the way to the summer of 2020…almost three months into the pandemic. And it was before I started my first year of medical school. We had just started to get to know our class… George Floyd had just been murdered and there was increasing news about COVID disproportionately affecting minorities. And it started being more about everything that’s wrong with the medical system, everything that’s wrong with systemic racism in America, all of it. I think it was a really high point in that people were really tired. (10)*

In terms of their profession, these topics were viewed as timely and important given that as future health care providers, they could end up treating victims of racially-motivated violence or patients who face difficult odds of surviving infectious diseases due to social determinants of health.

Despite the importance of racial justice protests in 2020 for the participants in this study, some students also expressed concern that less media attention on social justice issues since then has decreased the momentum needed to fuel positive changes. In terms of medical education, they worried that medical schools will feel less pressure to change policies and curricula. One student explained:

*The times kind of changed. Back in 2020, maybe a little bit of 2021, there was more of a movement towards social justice and just speaking up about everything. And now that’s kind of been toned down. A lot of medical schools are very conservative and they don’t really want to talk about racism or social inequities that openly. So, it’s kind of come back to that a little bit. (18)*

Similarly, some participants emphasized current challenges for medical education in the wake of critical race theory controversies and potential government restrictions on higher education, including censorship of topics labeled as “divisive”. Students’ questioning of how politics could potentially affect the inclusion of social inequalities in formal medical curricula also connects with their overall concerns about power structures. While students viewed the class as empowering, limitations on their agency are also an important part of this theme. Students perceived their own lack of power given their low-status positions in the hierarchical medical system, and expressed fears that disrupting the status quo might lead to punishments (accusations/complaints regarding their “professionalism”) that could jeopardize their progress and future opportunities. As such, they discussed the need for strategies to work within the constraints they faced as students now and as they move through their training and careers.

### Theme 3: creating community

The last theme regarding students’ motivations for participating in the elective focuses on their desires to create a community of learning and practice with people who have shared worldviews, values, and goals. Students expected that the class would facilitate their educational and networking goals by fostering connections among like-minded individuals and offering a safe space to discuss complex topics. The following two comments are representative of the kind of class environment students hoped for in the elective.

*It’s nice to be in a room with like-minded people. Not necessarily an echo chamber because it’s not an echo chamber. Definitely different opinions in there, but just people who can talk about those topics comfortably and respectfully, too. (20)*

*I was really interested in just educating myself about everything going on in the United States and just having a safe space to even like vent with my classmates as well. (17)*

Because the class was optional, students assumed that other classmates who chose to participate would share their interests in medical racism and health inequities. A mandated class might not bring the same kinds of advantages, in terms of the supportive environment and potential for peer-to-peer learning during discussions. In this way, the course offered a space that was not otherwise available. The excerpt below illustrates this perspective.

*I think one of my personal goals was to facilitate dialogue amongst medical students. I feel like one of the more frustrating issues being in graduate academia, especially one that’s burdened by the constraints of “professionalism” is that people are afraid to engage in conversations that they might feel are inflammatory or incendiary. And so, I wanted to help provide a space where that can happen on the campus in a way that was still academic and controlled and didn’t resort to political hodgepodge. (2)*

This type of learning space was particularly important in light of the challenges of working toward health equity discussed in the previous, “enacting change” theme. A community of mutual support was necessary given the size and complexities of the problems students wanted to address. In the excerpt below, a student shares this view.

*I think in a context like medical school, we just need a space where students can come and learn from evidence based, peer-reviewed sources that say these are the facts. This is the evidence. This is how it is. These are the ways we can fix it that have been tested, and this is how you can implement change for your neighbors and how you can improve the systems that you occupy… It’s like, we just need more collaborators, we need more comrades. (7)*

The class offered a way to build not only a professional network, but also social connections. The example below illustrates this point.

*I thought it would be a good opportunity to learn and then also to talk with like-minded people. So, I was really excited both about the learning but then also about other, more social aspects. (12)*

Feeling connected to peers during medical education for social/emotional well-being was important to participants. Afterall, medical school was often overwhelming, difficult, and stressful. In some cases, students had already started to establish this connectedness with peers in “the community” and described learning about the class through their friends or involvement with student organizations. Others, however, were still searching for a sense of belonging and finding their “place” at the university. In the excerpt below, a student described their struggle after experiencing the fallout from a conflict during a discussion of racism with a classmate earlier on in their studies.

*I really had kind of shut down, like I didn’t talk at all anymore… I stopped participating even during review sessions. I really only talked to two classmates that I knew. (9)*

Although this student was apprehensive about engaging with their classmates again, they decided to participate in the elective course and emphasized how important that decision was for improving their personal mental health. An underlying finding is that both minoritized and privileged students in this group often felt isolated or at the social margins and struggled with fitting in during their training. Enrolling in the course was one way to potentially improve their social situation and overall well-being.

## Discussion

Our themes of pre-existing conditions, enacting change, and creating community contribute to adult learning theory by providing a deeper understanding of learner motivations for participating in an elective course focused on health inequities. Previous scholarship has emphasized the utility of applying Self-Determination Theory (SDT) (Deci & Ryan, 2000) to improve student motivation, and therefore educational outcomes, during the design and implementation of learning activities in medical education (Williams et al., 1999; Burgess and Ramsey-Stewart, 2014); and our findings demonstrate that SDT can also help explain why students choose to participate in a particular learning activity in the first place. Specifically, we interpret our results as showing that the psychological needs for autonomy, competence, and relatedness (Deci & Ryan, 2000) underlied students’ decisions to voluntarily take the elective.

First, our themes highlight the importance of autonomous motivations for enrolling in the elective course. Students chose to study systemic health inequities because they found the topics interesting and important. For instance, only two students mentioned that the course was a way to build a stronger resume (i.e., extrinsic motivation). Instead, participants emphasized how their interests developed in relation to their own “pre-existing conditions,” including their identities and previous personal, work, and educational experiences. Prior to the course, they had internalized views that the medical system is biased and perpetuates health disparities. Furthermore, their beliefs that health inequities are unjust motivated their desire to learn strategies for enacting change. In other words, they were not only driven to learn for learning’s sake, but for instrumental reasons (e.g., to provide quality care to diverse patients, make improvements in the larger medical field). These findings also align with Knowles’ (1980, 1984) points about adult learners as internally motivated, informed by life experience, and application-oriented.

While students had some previous understandings of these topics, self-assessments of their knowledge gaps and the need for competency also motivated their participation. They expressed wanting to gain expertise by learning more about medical history and social determinants of health (i.e., “facts” about medical racism and systemic health inequities) and developing the skills necessary to become effective health care providers. They framed the rewards of gaining competency not in individual terms, but rather for the benefit of patients, medicine, and society in general. They autonomously chose to engage in the course because the anticipated benefits of “competency” were consistent with their personal values and goals. In contrast to their other classes, they were not participating out of obligations to complete a required curriculum or to perform better on a board exam. In fact, their views that the formal curriculum alone would not adequately prepare them with the level of competency they desired motivated their enrollment in the elective.

The importance of relatedness, the sense of belonging or feeling connected to others (Ryan and Deci, 2000) is also evident, specifically in the theme of “creating community.” Students viewed the class as a way to meet and engage in a collaborative way with others who had similar values and interests. As such, the class was an opportunity to create or join a “community of practice” (Wenger, 1999). The class learning community provided a “safe space” for sharing ideas and experiences; and students frequently discussed how they hoped their peers and lecturers could foster changes in their own attitudes and perspectives (i.e., transformational learning). By transforming their personal outlooks and understandings, students could then work to transform medicine and society in positive ways (i.e., the community enables the learning necessary to “enact change”). In this community, transformative learning can then move from an individual-centered phenomenon to a social-emancipatory process (Freire, 2020) As learners are “constantly reflecting and acting on the transformation of their world so it can become a more equitable place for all to live,” this process leads to “social transformation by demythicizing reality” (Taylor, 2008: 8).

Not only were their relationships with other students, educators, and professionals in the class important to participants, but the larger institutional environment was also key to their decisions to enroll in the elective. In addition to their assessments of what they were learning in the mandatory curriculum, students’ appraisals of faculty expertise and comfort discussing complex topics such as racism and other social inequalities, behavioral expectations of students (“professionalism”) in the college, and the overall culture of medical school motivated their participation in the course. Beyond the institution, the finding that participants were inspired by social movements further indicates the importance of the larger social and political context for motivating students to take advantage of learning opportunities that are relevant to their field. Events going on in the world, from protests to pandemics, influence the pursuit of adult education (Belzer & Dashew, 2023; Walker & Butterwick, 2020), as adults connect their day-to-day informal learning with formal learning opportunities. Students’ exposure to protests and media reports focused on the outcomes of violence and other forms of oppression contributed to their developing worldviews and compelled them to learn more in order to “enact change.”

Given that students discussed having multiple motivations for participating in the elective, a model that describes participation as a chain of responses to multiple factors (Cross, 2003) aids interpretation of results. Students’ self-evaluation of their knowledge and abilities as well as their desire for accurate information figured prominently in the “pre-existing conditions” theme. Similarly, their attitudes toward education were framed by their previous experiences, backgrounds, social group influences, and assessments of the mandatory curriculum and instruction. Many participants described life transitions during which they became aware of inequalities in personal, firsthand ways (e.g., attending college in a diverse city, working with unhoused people in a job after college). They had goals of becoming competent health care providers and contributing to large-scale improvements in medicine and society. Students expected that participation in the course would help them meet their goals, based on the design, format, content, and peer participation in the class. In addition to their two career-focused goals, they viewed the class as an opportunity to meet and network with “like-minded” people (the possibility of “creating community”). In the end, the anticipated benefits outweighed the barriers to participation such as the additional time demand, balancing the course with their other obligations, and potential discomfort discussing difficult topics.

Few studies have examined medical students’ reasons for participating in optional training focused on medical racism and systemic health inequities. However, many of findings in the themes created for this case study relate to motivations and goals described in other contexts, including scholarship from medical student-authors advocating for curriculum development focused on anti-racism and social determinants of health in medical education. For example, our results provide further support that the following perceptions are widespread among medical students: medicine is a biased system that contributes to inequities (Nieblas-Bedolla et al., 2020; Afolabi et al., 2021), the current use and discussion of race in curricula are imprecise and inadequate (Nieblas-Bedolla et al., 2020; Afolabi et al., 2021; Lynn et al., 2023; Bright & Nokes, 2019), academic competence is important (including familiarity with academic literature and research evidence) (Gonzalez et al., 2020; Lynn et al., 2023), interdisciplinary approaches and inclusion of scholars from other fields, nonphysician instructors, and community members would benefit training/education (Nieblas-Bedolla et al., 2020; Afolabi et al., 2021; Gonzalez & Bussey-Jones, 2010), practical applications should be prioritized (such as providing effective care for patients from different backgrounds to mitigate health disparities and advocating for patients) (Gonzalez & Bussey-Jones, 2010; Gonzalez et al., 2020; Leung et al., 2016; Nieblas-Bedolla et al., 2020; Lynn et al., 2023), social movements and medical students’ roles in activism are significant for medical education (Afolabi et al., 2021), and a safe and collaborative discussion environment is essential to learning (Gonzalez & Bussey-Jones, 2010; Gonzalez et al., 2020; Leung et al., 2016; Lynn et al., 2023).

Our themes offer more insight into the points on this list, particularly in terms of understanding how and why students develop these views, the role of power structures in students’ experiences during medical school, and the ways in which students seek out learning environments to achieve educational and social goals. For instance, previous studies recognize that personal interest motivates students to participate in voluntary learning activities, and that self-selected learners often have more knowledge prior to the training (Lynn et al., 2023; Bright & Nokes, 2019); however, these “pre-existing conditions” are not explored further, but instead discussed mostly with regard to how they complicate evaluation due to selection bias (i.e., less opportunity to show growth pre and post intervention). On the other hand, our analysis indicates that students developed their knowledge and interest through specific personal, educational, and work experiences during their lives. Although students in the Lynn et al. (2023) study described their lack of lived experiences with racism as a barrier to discussion participation due to inadequate confidence, students in our study discussed their lack of lived experience differently, framing it as a reason why they needed to engage with others who did have relevant personal experiences in order to learn more. All of the students in our sample identified their personal backgrounds and experiences as motivating factors for participation, which is a new finding that deserves attention. In particular, we see how quite varied experiences can motivate students from minoritized and privileged groups to participate in a medical elective focused on social inequality.

Similarly, our analysis of “creating community” delves deeper into students’ preferences about learning environments. Beyond seeking a “safe” space to learn, students viewed the class as a way to identify people with similar values and establish not just professional connections, but also social bonds with their peers. We expect that this motivation was especially important because of the elective topic and the particular students who self-selected to participate in the class. If they felt socially alienated during medical school because of their identity or beliefs about social inequality and medicine, their need for relatedness was likely high. Students anticipated that the potential mutual support, both personally and professionally, would help them cope with their frustrations, overcome challenges, and feel included. By providing opportunities for them to strategize with others about how to confront and resist power structures while engaging in “self-preservation” to enable their successful progression through medical school, this community was empowering for students. It is also important to note that while some of their views of medical school culture and curricula were critical, they demonstrated a genuine interest in helping to address the problems they identified. In this way, students can be viewed not as “trouble-makers” but rather as potential collaborators for academic leaders and educators who are similarly committed to improving medical education (also, see Könings et al., 2021).

### Limitations

Participation in the study was voluntary. The themes reflect the perspectives of medical students who were not only motivated to enroll in the course, but also motivated to participate in the research. As such, there is a possibility that the study does not include every major factor that influenced students’ decisions to participate in the elective. Also, there may have been differences between participants and non-participants that could have influenced the results (e.g., study participants may have had more prior knowledge about social inequalities than non-participants). Research participants reported an interest in engaging in the interview in order to reflect further on what they learned in the class, support future course development at their university, and contribute information that might aid larger-scale efforts to build curricula focused on systemic health inequities at other schools. Although the sample was small and from one institution, the results do align with and build on (the limited) previous research in other contexts. It is also important to note that this study included primarily first and second year medical students (and only one third year student). Although we did not observe differences based on participant’s year across our themes, motivations to engage in learning activities likely vary over the course of medical training. Future research is needed to identify potential changes in motivation, especially in light of evidence that students experience “empathy fatigue” during the third year of medical school (Hojat et al., 2009).

### Applications

The findings of this study can be applied, at this university and beyond, to support effective educational interventions including new course development, design, and instruction, especially with regard to elective options. Although voluntary courses do not reach as many students as required program components, students who choose to participate are actually invested in the topic and committed to creating a supportive learning community. As autonomously-motivated learners, students are likely to foster greater reflection and peer engagement in the classroom (Azila-Gbettor, 2021; Sobral, 2004). In our study, many participants noted that the course material and topics were appropriate for the mandatory curriculum; however, they appreciated the elective format because the other self-selected students were more likely to be “like-minded” and interested in creating a “safe space” for high level dialogue. As such, we draw attention to the advantages of an elective format– students with autonomous motivation and community-building potential– in order to encourage educators to consider these benefits when they design curricula.

With regard to developing elective learning activities, we also suggest keeping the following findings in mind. Medical students may have foundational knowledge about social inequities, but many desired more expertise on how these topics relate to medicine, specifically. This supports previous literature that also shows a strong student demand for education about racism, bias, social inequities, and health disparities to supplement current curricula. When offered, these programs and courses are likely to attract diverse students with varied experiences to draw on in discussions; and students who enroll are eager to hear their classmates’ and facilitators’ perspectives that differ from their own. As such, courses should be discussion-based to facilitate peer-to-peer learning. Course content should also include attention to contemporary issues in the news and other media that relate to health and health care, including current social movements that the students are exposed to in their daily lives and find important.

Given that students emphasized application-oriented goals/expected benefits in their responses, courses should also focus on providing strategies that students can implement during their clinical training and future practice that help them address problems of equity in health and health care, provide a higher quality of care to patients of varied backgrounds, and work towards larger positive changes in medicine and society. Because students also stressed the need for experts in the roles of course presenters and discussion facilitators, we suggest that medical curriculum committees reach out to scholars and practitioners in a variety of fields, such as history, political science, law, sociology, and anthropology, as well as professionals in the community who have the training and qualifications to provide accurate information and support effective learning about these topics.

Although we can make some initial recommendations based on this study, additional research about learners’ motivations and experiences, ideally across differently-formatted learning opportunities, is needed to create effective models for education focused on anti-racism and systemic health inequities, including the establishment of best practices in mandatory and optional training. For instance, some formats, like the course described in this study, may work most effectively with self-selected learners, while other learning activities should be mandatory. Additional research could help clarify how to balance the need to reach as many students as possible with providing more in-depth training to interested students, given that outcomes likely depend on students’ motivations. Similarly, creative methods for evaluating elective courses are needed since learners are likely to begin the class with more interest and knowledge about the topic (i.e. ways to solve the issue of “selection bias”).

## Conclusion

*People say the universe arches towards justice, right? But for me, it’s like it arches because people are pushing for it. It must be pushed in the correct direction. And people sacrifice a lot…Women today can go into medicine because people protested. They went out on the streets. It’s not like the people who were legislating had a sudden change of heart…You always need to pressure people, you know, and so you need to push in your own way to do these things regardless of how much work it takes. So, you’ve got to do it, if you want to see the change. You can’t wait for other people to sacrifice their time. You have to do it. That’s my belief at least. (8)*

Our interpretations of participants’ motivations through the themes of pre-existing conditions, enacting change, and creating community are intended to help academic leaders and medical educators understand students’ viewpoints in a comprehensive way. Adult learning theory and Self-Determination Theory emphasize the importance of including learners’ goals and needs into decisions about educational programs (Könings et al., 2021). As such, we also advocate for the inclusion of student voices in discussions about curriculum development and design, especially in light of larger political debates about appropriate training and presentation of complex topics like systemic racism in higher education. As evident in the students’ perspectives described here, they make their own cases for why instruction about medical racism and systemic health inequities is essential to their medical education and their future careers as competent physicians. In addition, establishing effective educational models requires attention to what kinds of benefits can be gained from both voluntary and mandatory training formats. Given that required and optional activities have different kinds of advantages, perhaps making more space for both would improve medical education.
